# Plasma insulin-like growth factor-binding protein 7 predicts 20-year cancer-specific and overall survival in the Atherosclerosis Risk in Communities Study

**DOI:** 10.1093/jnci/djag238

**Published:** 2026-07-15

**Authors:** Vernon A. Burk, Michael N. Pollak, Corinne E. Joshu, Anna Prizment, Elizabeth A. Platz

**Affiliations:** 1 Department of Epidemiology, Johns Hopkins University, Baltimore, MD, United States; 2 Lady Davis Research Institute, Jewish General Hospital, McGill University, Montreal, QC, Canada; 3 Sidney Kimmel Comprehensive Cancer Center at Johns Hopkins, Baltimore, MD, United States; 4 Department of Laboratory Medicine and Pathology, University of Minnesota Medical School, Minneapolis, MN, United States

## Abstract

**Background:**

Insulin-like growth factor-binding protein 7 (IGFBP7) may influence physiology by modulating IGF signaling and/or by IGF-independent mechanisms. Prior studies suggest positive associations of circulating IGFBP7 with cancer incidence and all-cause mortality. We analyzed whether IGFBP7 is associated with cancer incidence, cancer mortality, and all-cause mortality.

**Methods:**

We conducted a prospective cohort analysis of 10 834 participants with no cancer history for cancer analyses, and 11 761 participants for all-cause mortality analyses in the Atherosclerosis Risk in Communities study. Participants aged 46-70 years were followed for >2 decades. Plasma IGFBP7 was measured by SomaScan 5K assay (relative fluorescence units). Incident cancers were ascertained primarily by state cancer registry linkage and mortality confirmed by death certificates. We estimated the association between quartiles of log_2_-transformed IGFBP7 and outcomes using Cox proportional hazards regression adjusting for age, sex, race/center, and major risk factors, and predicted 20-year survival across IGFBP7 quartiles.

**Results:**

IGFBP7 was positively associated with cancer mortality (1420 cancer deaths; Q4 vs Q1: HR = 1.27, 95% CI = 1.08 to 1.50), especially lung (HR = 1.48, 95% CI = 1.07 to 2.05), but not with cancer incidence (3347 cases; HR = 1.01, 95% CI = 0.91 to 1.12). IGFBP7 was positively associated with all-cause mortality (6981 deaths; HR = 1.33, 95% CI = 1.23 to 1.43). The highest IGFBP7 quartile had the lowest 20-year predicted cancer-specific survival (unadjusted model only) and survival overall. IGFBP7 was not strongly correlated with IGF-1.

**Conclusions:**

High circulating IGFBP7 may be a risk factor for cancer mortality, especially lung, and all-cause mortality. Our observations justify confirmatory studies and efforts to understand underlying mechanisms.

## Introduction

A Women’s Health Initiative (WHI) study reported that plasma insulin-like growth factor-binding protein 7 (IGFBP7) is associated with total and obesity-associated cancer incidence and all-cause mortality.^1^ A study in PREDICTOR of older adult men and women reported a positive association for IGFBP7 with all-cause mortality.^2^ Scans of an Olink panel of >1000 proteins in the UK Biobank identified IGFBP7 as positively associated with all-cause mortality adjusting for risk factors,^3^ but not with risk of major cancers.^3,4^

IGFBP7 is a secreted protein with sequence homology to other insulin-like growth factor-(IGF) binding proteins, and as such, is expected to prevent IGF-1 from signaling through its receptor. This purported function is important because plasma IGF-1 is positively associated with epithelial cancers.^5–7^ However, IGFBP7 differs from other IGF binding proteins as it has a relatively low affinity for IGF-1 and IGF-II,^8^ and interacts not only with IGF ligands but also with the IGF-I receptor,^9^ and with other molecules including CD93,^10^ which is involved in capillary integrity and potentially with metastasis. Physiologic functions of IGFBP7 are incompletely understood. Known polymorphisms in *IGFBP7* account for only a small degree of the between-person variability in circulating levels of IGFBP7,^11^ and non-genetic factors contributing to this variability have not been rigorously investigated.

Given the potential for IGFBP7 to influence metastasis and that other studies did not address cancer mortality,^1–4^ we investigated the association of plasma IGFBP7 with cancer incidence, cancer mortality, and all-cause mortality, in the Atherosclerosis Risk in Communities (ARIC) study, a cohort of mostly White and Black, women and men. We also assessed whether IGFBP7 measured at various ages in adulthood predicts 20-year risk of cancer mortality and all-cause mortality.

## Methods

### Study population

ARIC is a prospective cohort of 15792 participants recruited in 1987-1989 from 4 centers: Forsyth County, NC; Jackson, MS; suburban Minneapolis, MN; and Washington County, MD.^12^ Only Black participants were recruited in Jackson, MS. The male and female participants were aged 45-64 years; 99.7% were Black or White. They attended multiple subsequent visits. We used Visit 2 (baseline) proteomics data. We excluded participants who were not Black or White (*n* = 48) and then excluded those with missing IGFBP7 data at Visit 2 (*n* = 3983: 318 deceased, 1120 did not attend Visit 2, 2545 did not have sufficient remaining plasma); 11761 participants comprised the all-cause mortality analyses. For cancer analyses, we additionally excluded participants diagnosed with cancer before Visit 2, yielding 10834 participants. Participants provided informed consent. Institutional review boards at the centers approved the ARIC study protocol.

### IGFBP7 measurement

Plasma IGFBP7 was measured using the SomaScan 5K assay at Visits 2 (1990-1992), 3 (1993-1995), and 5 (2011-2013). We evaluated 4712 proteins detected by 4955 aptamers passing quality control.^13^ Protein levels in a standard plasma volume are expressed in relative fluorescence units (RFU). Levels were preprocessed using adaptive normalization maximum likelihood (ANML) standardization and log_2_-transformed. The intrapair CV% for IGFBP7 was 5.1% for Visit 2 and 5.4% for Visit 5 in a prior ARIC sub-study.^14^

### Outcome ascertainment

Incident cancer diagnoses were ascertained via linkage with cancer registries in MD, MN, MS, and NC, supplemented with medical records, hospital discharge summaries, and death certificates.^15^ Deaths were identified from death certificates, participant informants, local newspaper obituaries, and National Death Index linkage.^12^ Cancer deaths were identified from death certificates indicating cancer as the underlying cause among those without a cancer diagnosis at Visit 2.^15^

### Covariate assessment

We used covariate data from Visit 2, unless otherwise specified. We used information collected by interview on age, sex, race and center, education (Visit 1), smoking status, packyears, alcohol-drinking status, and hormone replacement therapy use (HRT, females). Diabetes status was categorized as diagnosed (self-reported physician diagnosis or diabetes medication use), undiagnosed (fasting glucose ≥126mg/dL, non-fasting ≥200mg/dL), at-risk (fasting glucose 100-125 mg/dL, non-fasting 140-199 mg/dL), or normoglycemic. BMI (kg/m^2^) was calculated using height and weight measured by trained staff. Hypertension was defined as diastolic blood pressure ≥90mmHg measured by trained staff or use of any anti-hypertensive medication assessed by review of the bottles of all prescription and over-the-counter medications taken in the last 2 weeks. Plasma total, high (HDL), and low-density lipoprotein (LDL) cholesterol were measured as part of the study protocol. We used a smoking-related plasma protein score that we generated, which is associated with lung cancer risk independent of smoking,^16^ and the Sathyan proteomics aging clock, which was trained in older adults and used the same proteomics platform.^17^

### Statistical analyses

Analyses were performed using SAS v9.4. Tests were 2-sided with *P* < .05 considered statistically significant. We estimated age-adjusted characteristics by IGFBP7 categories using linear regression. Cox proportional hazards regression was used to estimate HR and 95% CI for the association between IGFBP7 (cohort quartiles, per doubling) and cancer incidence, cancer mortality, and all-cause mortality. We ran minimally (Model 1: age, race/center, sex) and fully adjusted models (Model 2: age, race/center, female/not current HRT user, female/current HRT user, male; education; smoking; packyears; diabetes; BMI; alcohol-drinking; hypertension; HDL-cholesterol). Other outcomes were incidence and mortality from common cancers: post-menopausal breast, prostate, lung, colorectal. Participants contributed person-time from Visit 2 to outcome, death, or December 31, 2015 for cancer analyses, and December 31, 2020 for all-cause mortality analyses. We tested for trend by entering a term for IGFBP7 quartile ranks and using the Wald test. To address residual confounding by smoking in lung cancer analyses, we additionally adjusted for the smoking-related protein score.^16^ We stratified by age quartiles, race, sex, HRT, and smoking status, and tested for interaction using the Wald test. We report the estimates for quartiles as the primary results, and for efficiency, report per doubling estimates for stratification and sensitivity analyses.

We assessed whether IGFBP7 provides risk information about mortality in short, medium, and long-terms. First, we ran the Cox model truncating follow-up at 1995 (3-5 years after blood collection), 2000 (8-10 years), 2005 (13-15 years), and 2010 (18-20 years). Second, we restricted to 4221 participants with protein data at all 3 visits, started follow-up at Visit 5 and ended follow-up at 5years after Visit 5, and estimated the association between IGFBP7 and all-cause mortality sequentially using protein data from each visit; we could not run this analysis for cancer mortality due to few events after Visit 5. Third, using IGFBP7 quartile cutpoints separately defined for each Visit 2 age quartile, we estimated 20-year survival overall and by age quartiles using the baseline statement in PROC PHREG.

To address reverse causation, we used Visit 2 IGFBP7 and lagged the start of follow-up to 5 and 10years later. Given a prior ARIC finding from a proteomics scan that IGFBP7 is associated with heart failure,^18^ for all-cause mortality we ran analyses censoring at incident heart failure. To assess whether IGFBP7 captures effects of other proteins, we calculated Spearman correlations with each panel protein. For the 10 proteins most strongly correlated with IGFBP7 (*r*≥ |0.34|), we adjusted for them in Model 2.

Because IGFBP7 may be involved in aging and senescence,^19–21^ we assessed IGFBP7’s independence from the Sathyan clock.^17^ We calculated the Spearman correlation and performed residuals analysis by regressing IGFBP7 on the clock using linear regression adjusting for covariates from Model 2 and entering IGFBP7 residuals with the clock in Model 2.

## Results

We ascertained 3347 first primary cancer cases in 200279 person-years (median follow-up 22.8years), 1420 cancer deaths in 222320 person-years (23.5 years), and 6981 deaths in 262734 person-years (25.6 years). Mean age was 57.1 years, 55.6% were female and 23.6% were Black. Age was weakly correlated with IGFBP7 (*r* = 0.07, *P* < .0001). After age-adjustment, participants in the highest IGFBP7 quartile vs lowest were more likely to be male, Black, current smokers, and have hypertension, diabetes, lower HDL cholesterol, higher BMI, and higher packyears of smoking. In addition, current HRT use is substantially more prevalent in the lowest quartile than the other 3 quartiles (Table 1, Table S1).

### Cancer incidence

In the minimally adjusted model (Model 1), IGFBP7 was positively associated with cancer incidence (Table 2), specifically lung (Table S2). However, additionally adjusting for risk factors (Model 2), IGFBP7 was not associated with cancer incidence (Q4 vs Q1: HR = 1.01, 95% CI = 0.91 to 1.12) Associations did not differ by age, race, or sex (Table 2), or HRT use (Model 2, per doubling, users: HR = 0.96, 95% CI = 0.65 to 1.42; nonusers: HR = 1.09, 95% CI = 0.82 to 1.45). For lung cancer, the association was attenuated in Model 2, and when further adjusting for the smoking-related protein score (Table S2).

### Cancer mortality

IGFBP7 was positively associated with cancer mortality in Model 1 (Q4 vs Q1: HR = 1.59, 95% CI = 1.35 to 1.86) and Model 2 (HR = 1.27, 95% = CI 1.08 to 1.50) (Table 3). In Model 1, IGFBP7 was associated with breast and lung cancer mortality (Table S3). In Model 2, IGFBP7 was associated only with lung cancer mortality, although attenuated (Q4 vs Q1: HR = 1.48, 95% CI = 1.07 to 2.05). Further adjusting for the smoking-related protein score, the lung cancer mortality HR was consistent (HR = 1.42, 95% CI = 1.02 to 1.97) (Table S3). The cancer mortality HR was only modestly attenuated when censoring lung cancer mortality (Q4 vs Q1: Model 2: HR = 1.21, 95% CI = 1.00 to 1.47).

Stratifying by age, the strongest association was in the youngest age quartile (46.9-52.5 years; Model 2, per doubling: HR = 1.97, 95% CI = 1.05 to 3.68) (Table 3). The association did not differ by sex (*P*-interaction = .80) or race (*P*-interaction = .94) (Table 3). Stratified by smoking status, the association with cancer mortality was stronger in current smokers (22.4% of the cohort; Model 2, per doubling: HR = 1.70, 95% CI = 1.13 to 2.56) and was in the positive direction, albeit not statistically significant in former (39.0%; HR = 1.17, 95% CI = 0.79 to 1.75) and never smokers (38.6%; HR = 1.28, 95% CI = 0.80 to 2.05). For lung cancer mortality, the association for IGFBP7 was present in current smokers (256 lung cancer deaths; Model 2, HR = 2.14, 95% CI = 1.22 to 3.73; after further adjusting for the smoking-related protein score: HR = 2.25, 95% CI = 1.26 to 4.01), but not in non-current smokers (161 lung cancer deaths; Model 2, HR = 0.94, 95% CI = 0.44 to 2.01). The association was present in non-HRT users (Model 2, per doubling, HR = 1.51, 95% CI = 1.00 to 2.29) but null in current users (HR = 0.86, 95% CI = 0.45 to 1.66; *P*-interaction = .27). In users and nonusers, the most common cause of cancer death was lung (~22%). Breast cancer comprised 20.2% of cancer deaths in nonusers and 12.8% in users.

When follow-up was truncated at 5, 10, 15, and 20 years (Table S4), or lagged to start 5 and 10years after Visit 2 (Table S5), the association remained positive, and of the same magnitude.

### All-cause mortality

IGFBP7 was positively associated with all-cause mortality in Model 1 (Q4 vs Q1: HR = 1.64, 95% CI = 1.52 to 1.76) and Model 2 (HR = 1.33, 95% CI = 1.23 to 1.43). Although attenuated in Model 2, HRs increased across quartiles (*P*-trend < .0001, Table 4). The strongest association was in the youngest age quartile (46.9-52.6 years; Model 2, per doubling: HR = 2.52, 95% CI = 1.88 to 3.38). The association did not differ by race (*P*-interaction = .61). The association was stronger among females than males (*P*-interaction = .04). Stratified by smoking status, the association was stronger in current smokers (22.4% of the cohort; Model 2, per doubling: HR = 2.06, 95% CI = 1.67 to 2.55) and comparable to overall in former (39.0%; HR = 1.49, 95% CI = 1.26 to 1.78) and never smokers (38.6%; HR = 1.62, 95% CI = 1.35 to 1.95). Among females, we observed an interaction (*P*-interaction = 0.02) between HRT and IGFBP7, where the risk of all-cause mortality was lower in current users (Model 2, per doubling: HR = 1.44, 95% CI = 1.08 to 1.93) than in nonusers (HR = 2.00, 95% CI = 1.68 to 2.38).

The strength of association increased after truncating follow-up time every 5years shorter (Table S6), and remained positive in lagged analyses (Table S7). Excluding participants with prevalent or missing heart failure Visit 2 information, and censoring for incident heart failure, the association remained, but slightly attenuated (Table S8).

### Within-person stability over time

Pairwise Spearman correlations between plasma IGFBP7 levels were *r* = 0.57 for Visits 2 and 3 (3 years apart), *r* = 0.36 for Visits 3 and 5 (18 years apart), and *r* = 0.44 for Visits 2 and 5 (21 years apart); all *P* < .0001. When restricting to participants who had IGFBP7 measured at all 3 visits (*n* = 4221), the intraclass correlation coefficient was 0.44 (*P* < .0001).

### Timing of blood draw relative to older age, short-term all-cause mortality risk

**Figure 1.**
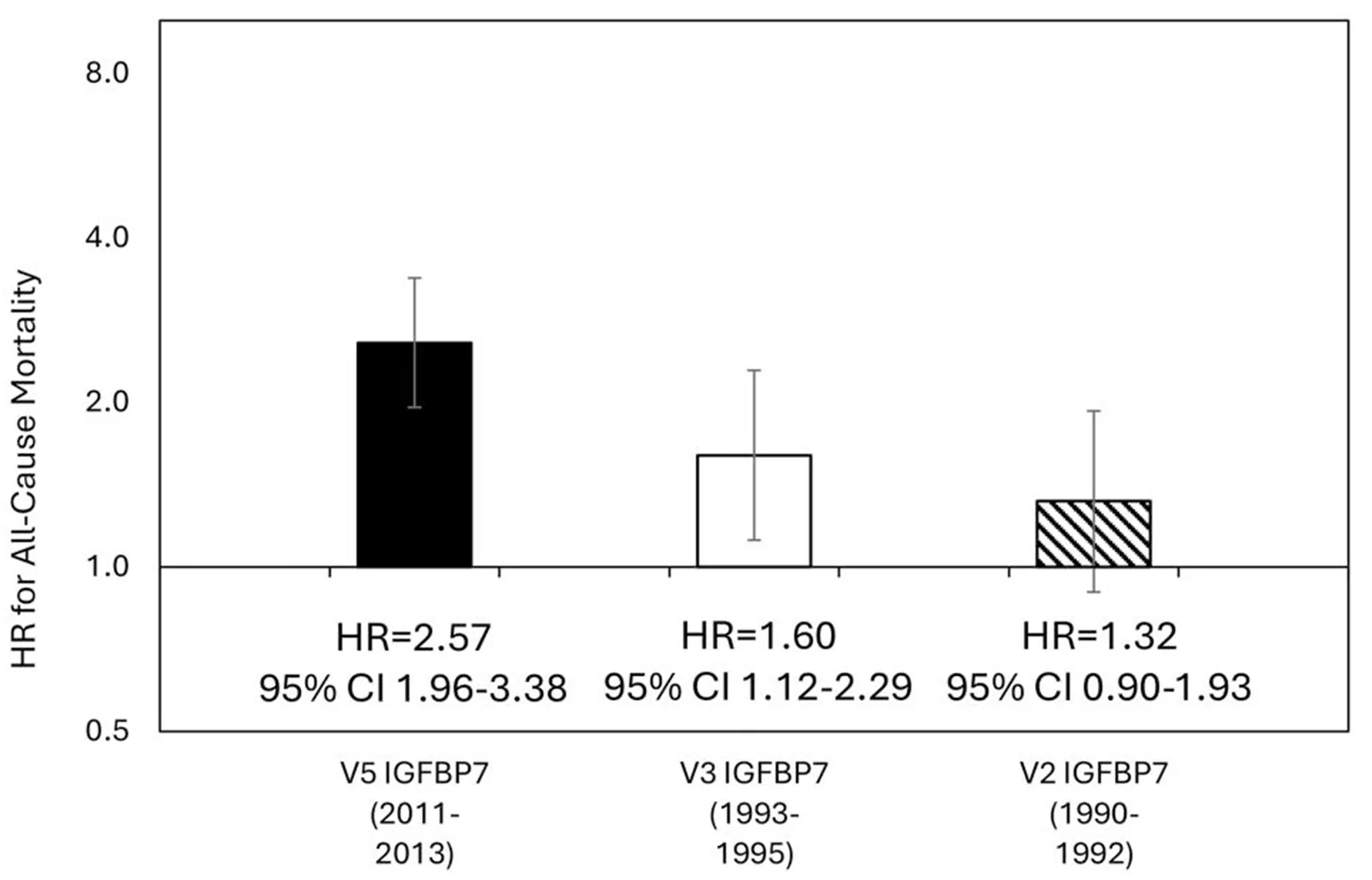
Association between plasma IGFBP7 and all-cause mortality, Visit 5 (2011-2013)—5 years of maximum follow-up; Cox proportional hazards models; includes only participants with levels at all 3 time points; same figure associated with Table S9. Using later life IGFBP7 (Visit 5), the association between IGFBP7 and all-cause mortality was strongest. Using earlier life IGFBP7 (Visit 3) from 18years before, and keeping follow-up time consistent starting at Visit 5, the association was attenuated but still statistically significant. Using earlier life IGFBP7 (Visit 2) from 21years before, and keeping follow-up time consistent starting at Visit 5, the association substantially attenuated. The models were fully adjusted for: age (continuous); race/center (Black/Jackson, MS, Black/Other County, White/Forsyth County, NC, White/Washington County, MD, White/Minneapolis, MN [ref]); female/not-current hormone replacement therapy user, female/current hormone replacement therapy user, male (ref); current smoker, former smoker, never smoker (ref); packyears (continuous plus a missing indicator); diagnosed diabetes, undiagnosed diabetes, at-risk for diabetes, normoglycemic (ref); BMI (continuous); at least some college, completed high school/equivalent, less than completed high school (ref) (Visit 1); current drinker, former drinker, never drinker (ref); hypertension status (yes/no); HDL cholesterol (continuous). Abbreviations: HR = hazard ratio (per doubling); V5 = Visit 5; V3 = Visit 3; V2 = Visit 2.

We assessed whether IGFBP7’s association with short-term all-cause mortality risk at older age is comparable when IGFBP7 is measured recently vs 2 decades in the past. We included participants with IGFBP7 measured at all 3 visits, began follow-up at Visit 5 when participants were ages 66-90, ended follow-up 5years later, and examined IGFBP7 measured at Visits 5, 3, or 2. For Model 1, the association was strongest using later life IGFBP7 (Visit 5), yet remained when measured 18 and 21 years before the start of follow-up. In Model 2, the association was still strong using later life IGFBP7 (Visit 5), was attenuated but statistically significant using levels from 18 years before, and substantially attenuated when using levels from 21 years before (Table S9, Figure 1).

### Predicted survival

**Figure 2.**
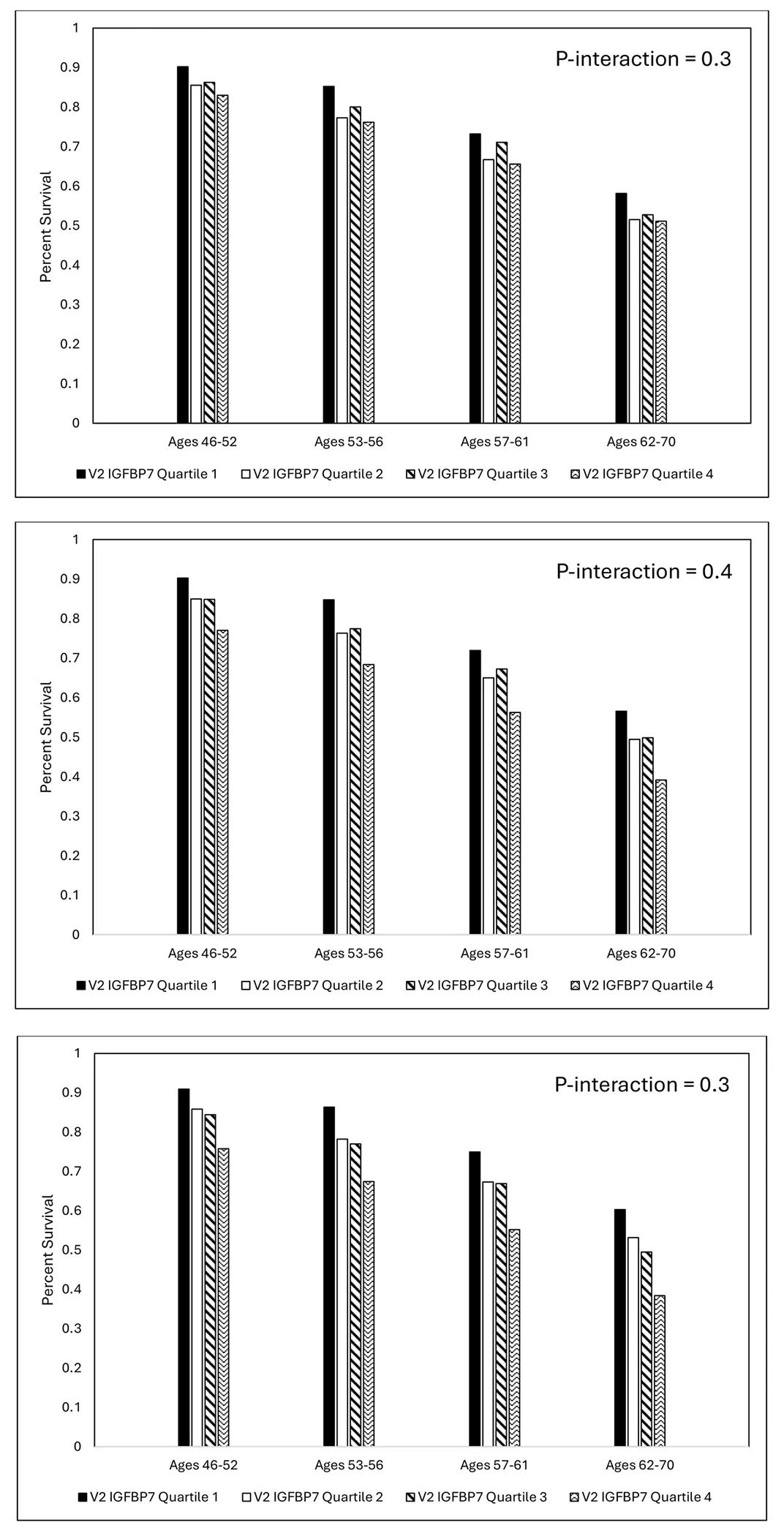
Overall survival estimates by age group and IGFBP7 quartiles, Visit 2 (1990-1992)—20 years of maximum follow-up; predicted survival (complement of all-cause mortality); Cox proportional hazards models. **A)** In the fully adjusted model, across each age quartile, the highest IGFBP7 quartile had the lowest probability of overall survival for all-cause mortality over 20 years. The model was adjusted for: age (continuous); race/center (Black/Jackson, MS, Black/Other County, White/Forsyth County, NC, White/Washington County, MD, White/Minneapolis, MN [ref]); female/not-current hormone replacement therapy user, female/current hormone replacement therapy user, male (ref); current smoker, former smoker, never smoker (ref); packyears (continuous plus a missing indicator); diagnosed diabetes, undiagnosed diabetes, at-risk for diabetes, normoglycemic (ref); BMI (continuous); at least some college, completed high school/equivalent, less than completed high school (ref) (Visit 1); current drinker, former drinker, never drinker (ref); hypertension status (yes/no); HDL cholesterol (continuous). **B)** In the minimally adjusted model, across each age quartile, the highest IGFBP7 quartile had the lowest probability of overall survival for all-cause mortality over 20 years. The model was adjusted for: age (continuous), race/center, sex (female/male). **C)** In the unadjusted model, across each age quartile, the highest IGFBP7 quartile had the lowest probability of overall survival for all-cause mortality over 20 years. The pattern is strongest in this model. Abbreviation: V2 = Visit 2.

**Figure 3.**
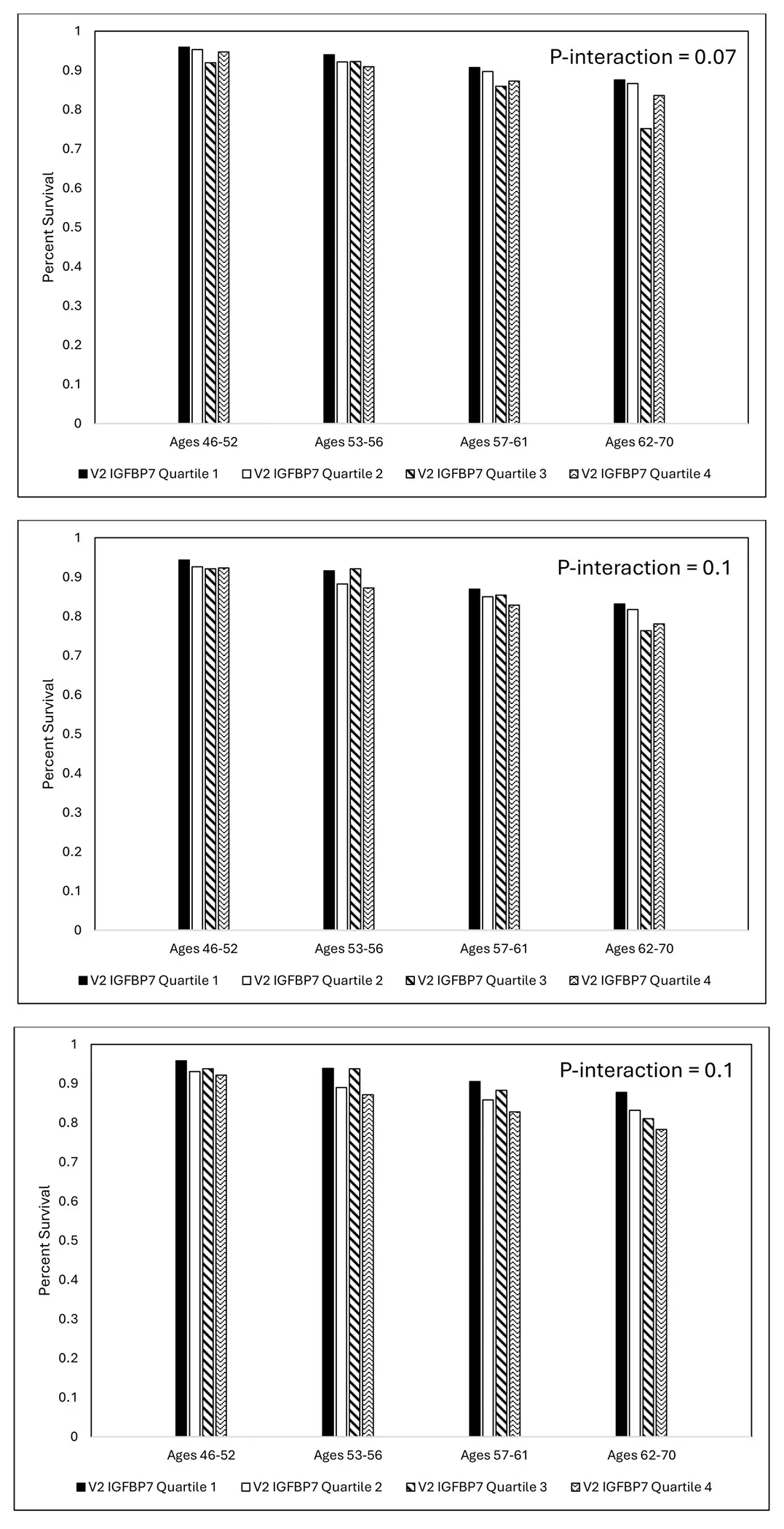
Cancer-specific survival estimates by age group and IGFBP7 quartiles, Visit 2 (1990-1992)—20 years of maximum follow-up; predicted survival (complement of cancer mortality); Cox proportional hazards models. **A)** In the fully adjusted model, across each age quartile, the highest or second highest IGFBP7 quartile had the lowest probability of cancer-specific survival over 20 years. The model was adjusted for: age (continuous); race/center Black/Jackson, MS, Black/Other County, White/Forsyth County, NC, White/Washington County, MD, White/Minneapolis, MN [ref]); female/not-current hormone replacement therapy user, female/current hormone replacement therapy user, male (ref); current smoker, former smoker, never smoker (ref); packyears (continuous plus a missing indicator); diagnosed diabetes, undiagnosed diabetes, at-risk for diabetes, normoglycemic (ref); BMI (continuous); at least some college, completed high school/equivalent, less than completed high school (ref) (Visit 1); current drinker, former drinker, never drinker (ref); hypertension status (yes/no); HDL cholesterol (continuous). **B)** In the minimally adjusted model, across each age quartile, the highest or second highest IGFBP7 quartile had the lowest probability of cancer-specific survival over 20 years. The model was adjusted for: age (continuous), race/center, sex (female/male). **C)** In the unadjusted model, across each age quartile, the highest IGFBP7 quartile had the lowest probability of cancer-specific survival over 20 years. The pattern is strongest in this model.

Across each age quartile, the highest IGFBP7 quartile had the lowest 20-year predicted overall survival, irrespective of whether the model was fully, minimally, or unadjusted (Figure 2). Within each age quartile, differences in predicted overall survival across IGFBP7 quartiles were greatest in the unadjusted model (Figure 2). For cancer-specific survival, the pattern was clearest in the unadjusted model (Figure 3).

### Correlations

IGFBP7 was not strongly correlated with IGF-1 (aptamer 1: *r* = −0.18, aptamer 2: *r*= 0.07) or other IGF-family proteins (*r* = |0-0.24|). The association for IGFBP7 and cancer mortality (Model 2, per doubling: HR = 1.32, 95% CI = 1.03 to 1.68) and all-cause mortality (HR = 1.57, 95% CI = 1.41 to 1.75) remained after adjusting for IGF-1 (aptamer 1). IGFBP7 was weakly correlated with the inflammation marker C-reactive protein (*r* = −0.09) and survival factor growth/differentiation factor-15 (*r* = 0.24). Of the SomaScan proteins, only Diablo homolog had a Spearman correlation > 0.5 (Table S10). The association for IGFBP7 and all-cause mortality remained after adjusting individually for the 10 most strongly correlated proteins; the greatest attenuation was <10% when adjusting for Diablo homolog (Table S11).

### Independence from a proteomic aging clock

IGFBP7 and Sathyan proteomic aging clock^17^ values were weakly correlated (*r* = 0.11). IGFBP7 residuals (Model 2, per 1-unit increase: cancer mortality HR = 1.31, 95% CI = 1.03 to 1.67; all-cause HR = 1.47, 95% CI = 1.32 to 1.64) and clock (per 1-unit increase: cancer mortality HR = 1.05, 95% CI = 1.03 to 1.07; all-cause HR = 1.12, 95% CI = 1.11 to 1.13) were independently associated with cancer mortality and all-cause mortality.

## Discussion

Our ARIC data provide evidence that high plasma IGFBP7 level is a risk factor for both cancer mortality, particularly lung, and all-cause mortality, especially when measured in middle adulthood. When fully adjusting for potential confounders, IGFBP7 was not associated with cancer incidence. Our findings were robust even after censoring incident heart failure, which was previously observed to be associated with high circulating IGFBP7 level,^22^ suggesting that this protein provides risk information for other major causes leading to death. As the mechanisms by which IGFBP7 influences cancer mortality or all-cause mortality are not certain, our findings motivate additional mechanistic experiments and epidemiologic studies of this protein, including identifying the etiologically relevant window for risk.

Aside from associations of IGFBP7 with heart failure^22,23^ and acute renal injury,^24^ in the few studies investigating plasma IGFBP7, associations of this protein with cancer risk^1,3,4^ and all-cause mortality^1–3^ have been observed. Orenduff et al. observed positive associations with total and obesity-associated cancers in women for IGFBP7 as a candidate measured with a targeted assay,^1^ and Papier et al. identified positive associations for liver cancer and multiple myeloma but not common cancers in a proteomic scan correcting for false discovery in the UK Biobank.^4^ In our study, IGFBP7 (candidate, proteomic panel) was not associated with cancer incidence in women or men or with common site-specific cancers, including obesity-associated–post-menopausal breast, and colorectal, adjusting for common risk factors. In our separate multicancer proteomic discovery study in ARIC,^25^ consistent with Papier et al.,^4^ we identified IGFBP7 as associated with liver cancer incidence adjusting for cancer risk factors and correcting for false discovery; we confirmed this association in an external cohort.^25^ Before risk factor adjustment, IGFBP7 was associated with cancer incidence, specifically lung. Numerous risk factors for cancer and other chronic diseases differed across IGFBP7 levels. We also noted higher levels in men and Black participants. Future studies should carefully consider these potential confounders.

We observed a positive association between IGFBP7 and cancer mortality, including after adjustment for major cancer risk factors. This association was the strongest when measured in early mid-adulthood compared with later adulthood. The association was robust when lagging or truncating follow-up time. While the association did not differ by race or sex, in women the positive association was present only in non-HRT users. HRT users were less likely to be in the highest IGFBP7 quartile, consistent with that observed in the WHI from baseline to 1year after starting estrogen plus progesterone or estrogen only HRT.^26^

Among the common causes of cancer death, the association was strongest and statistically significant for lung cancer mortality. The lung cancer association was present only in current smokers. We noted that IGFBP7 and packyears were positively associated. Thus, in addition to adjusting for self-reported smoking status and packyears, we adjusted for a smoking-related protein score^16^; the association was not further attenuated overall or when restricting to current smokers. The score does not include IGFBP7. Given effect modification by HRT, we confirmed that the proportion of lung cancer deaths was comparable between users and nonusers.

IGFBP7 is thought to play a role in angiogenesis and vascular remodeling,^27^ including in tumors,^8^ and may mediate microvascular injury in the lung.^28^ Whether our observation of a positive association between IGFBP7 and lung cancer mortality reflects increased tumor angiogenesis coupled with increased metastatic dissemination due to increased vascular permeability, or other mechanisms, remains to be determined.

With respect to all-cause mortality, our finding of a positive association in both men and women was consistent with prior studies using targeted assays or measured in a proteomics panel.^1–3^ The association was the strongest when measured in early mid-adulthood, and when measured closer in time to mortality. The association was comparable in Black and White participants, and the association was robust when lagging the start of follow-up. The association was present among never smokers, helping to rule out residual confounding by current smoking, the prevalence of which increased across quartiles of IGFBP7 level. We noted that the association was stronger in women than men; in women the association was stronger in nonusers than HRT users; and the association was comparable in HRT users and men.

We propose plasma IGFBP7 should be further investigated as a biomarker for predicting cancer-specific survival and overall survival, especially in early middle-adulthood, in clinical settings in which risk factor history is unavailable. We documented that predicted 20-year survival was the lowest in the top quartile of IGFBP7 irrespective of quartile of age before and after adjusting for risk factors. We also documented that IGFBP7 level measured 2 decades earlier among those who survived was associated with conditional survival when adjusting for age, sex, and race (but attenuated after adjusting for risk factors).

This study has many strengths. We performed an analysis of data from a prospective cohort that included women and men, and Black and White participants with long-term follow-up. Our study was larger than previous studies in which IGFBP7 was a candidate protein.^1,2^ We showed that the protein is not well correlated with other members of the IGF signaling pathway, including IGF-1, suggesting that the observed associations are independent of that pathway. We also showed that IGFBP7 is not highly correlated with most other proteins in the SomaScan panel, including a major inflammatory protein and a survival factor, suggesting that this protein may not simply serve as a surrogate for previously characterized biomarkers. Nevertheless, the protein most strongly correlated with IGFBP7 was Diablo homolog; expression and methylation of the encoding gene has been associated with lung cancer risk and outcomes.^29,30^ The association between IGFBP7 and lung cancer mortality was only modestly attenuated when adjusting for this protein, again indicating that this protein is not merely a surrogate for a previously identified risk biomarker. Finally, IGFBP7 is associated with age,^1^ and has been suggested to be a marker for aging and/or senescence.^19–21^ In this context, we confirmed that the protein was not highly correlated with a published proteomic clock^17^ and that IGFBP7 provides information about all-cause mortality beyond that proteomic clock.

This study has possible limitations. The ARIC study does not include younger adults or persons of ancestries other than White or Black. The assay we used provides relative rather than plasma concentrations of IGFBP7, and we do not know the extent to which the aptamer detects IGFBP7 bound to other molecules. Future confirmatory studies are needed that use targeted, quantitative assays.

In conclusion, our prospective cohort study contributes to the evolving evidence demonstrating that plasma IGFBP7 deserves study as a clinically relevant biomarker. Our findings support its role as a predictor of cancer-specific survival and overall survival in early middle-adulthood through older adulthood, independent of common risk factors. While further research is needed to uncover underlying mechanisms, we speculate that the association of high IGFBP7 levels with cancer mortality, but not cancer incidence, may relate to a higher risk of metastasis in individuals with high IGFBP7 levels, which is plausible in the context of roles of IGFBP7 on microvasculature.^8,10,27^

## Supplementary Material

supplementary material

Supplementary material is available at *Journal of the National Cancer Institute* online.

## Figures and Tables

**Table 1.** Baseline age-adjusted characteristics by plasma IGFBP7 quartile,^a^ ARIC, Visit 2 (*n* = 11761).

Characteristics	Plasma IGFBP7			
	Quartile 1 Mean RFU (range): 14.67 (13.48-14.82)	Quartile 2 Mean RFU (range): 14.90 (14.82-14.96)	Quartile 3 Mean RFU (range): 15.02 (14.96-15.09)	Quartile 4 Mean RFU (range): 15.24 (15.09-17.42)
Participants, No.	2940	2940	2941	2940
Age, mean (SE), y	56.5 (0.1)	57.0 (0.1)	57.3 (0.1)	57.5 (0.1)
Female, %	77.5	56.4	45.9	42.7
Black, %	20.1	23.9	24.6	25.5
Center, %				
Forsyth County, NC	27.5	26.0	25.3	27.1
Jackson, MS^b^	18.2	20.8	21.3	21.2
Minneapolis, MN	32.5	26.7	25.6	21.9
Washington County, MD	21.8	26.5	27.8	29.8
Education level, %				
Less than completed high school	17.2	20.1	22.6	24.3
Completed high school/equivalent	45.5	44.3	40.4	40.3
At least some college	37.3	35.6	37.0	35.4
Smoking status, %				
Current smoker	14.3	20.3	24.0	30.6
Former smoker	37.4	39.7	40.8	38.1
Never-smoker	48.3	40.0	35.2	31.3
Packyears (ever smokers), mean (SE), y^c^	23.5 (0.6)	26.4 (0.6)	28.7 (0.5)	33.2 (0.5)
BMI, mean (SE), kg/m^2^	27.5 (0.1)	27.9 (0.1)	28.1 (0.1)	28.2 (0.1)
Hypertension, %	26.4	27.9	30.8	34.3
Diabetes status, %				
Diagnosed diabetes	6.7	8.3	9.6	12.7
Undiagnosed diabetes	5.6	5.8	7.3	10.3
At-risk diabetes	43.8	51.6	50.7	48.7
Normoglycemic	43.9	34.3	32.4	28.3
Drinking status, %				
Current drinkers	56.4	56.4	58.5	55.8
Former drinkers	17.1	21.4	20.0	25.3
Never drinkers	26.5	22.2	21.5	18.9
Cholesterol, mean (SE), mg/dL				
Total	214.5 (0.7)	213.1 (0.7)	208.1 (0.7)	204.4 (0.7)
High density lipoprotein (HDL)	55.4 (0.3)	49.5 (0.3)	47.7 (0.3)	46.4 (0.3)
LDL	131.4 (0.7)	136.8 (0.7)	134.1 (0.7)	131.1 (0.7)
Aspirin use, %	53.7	50.0	49.2	51.5
HRT use (females), %				
Current user	46.3	18.5	15.4	11.6

a Visit 2 (baseline) quartiles defined in the cohort used in the all-cause mortality analyses. Standard errors (SE) are provided for age-adjusted means for continuous variables.

b Only Black participants were recruited in Jackson, MS.

c Of 7222 ever smokers, packyears could not be determined for 635 (8.8%). They are excluded from this table packyears estimates.

**Table 2.** Association between plasma IGFBP7 RFU and risk of incident total cancer, ARIC, Visit 2 to 2015.

	Cancer cases	Person-years	Model 1^a^ HR and 95% CI	Model 2^b^ HR and 95% CI
Quartile^c^				
1	755	53592	reference	reference
2	847	51110	1.03 (0.93 to 1.14)	1.01 (0.91 to 1.12)
3	860	49528	1.01 (0.92 to 1.12)	0.97 (0.87 to 1.07)
4	885	46045	**1.12 (1.01 to 1.24)**	1.01 (0.91 to 1.12)
*P*-trend			.09	.77
Per doubling	3347	200275	**1.18 (1.01 to 1.37)**	1.01 (0.86 to 1.19)
Stratified by age				
< 52.5y	706	56071	1.24 (0.87 to 1.76)	1.06 (0.73 to 1.53)
52.5 to ≤ 57.1y	788	53174	1.22 (0.90 to 1.66)	0.99 (0.71 to 1.38)
> 57.1 to 62.3y	916	48540	1.23 (0.93 to 1.61)	1.16 (0.87 to 1.56)
>62.3y	937	42491	1.06 (0.79 to 1.42)	0.87 (0.63 to 1.18)
*P*-interaction^d^			.75	.79
Stratified by race				
White	2550	154363	1.17 (0.99 to 1.39)	1.00 (0.83 to 1.20)
Black	797	45912	1.29 (0.95 to 1.77)	1.12 (0.81 to 1.54)
*P*-interaction^d^			0.18	0.19
Stratified by sex				
Male	1810	84092	1.12 (0.89 to 1.39)	1.00 (0.80 to 1.26)
Female	1537	116183	**1.31 (1.07 to 1.62)**	1.05 (0.84 to 1.32)
*P*-interaction^d^			.60	.44

Bold denotes statistically significant.

a Adjusted for age (continuous); race/center (Black/Jackson, MS, Black/Other County, White/Forsyth County, NC, White/Washington County, MD, White/ Minneapolis, MN [ref]); and sex (female/male). We adjusted for race/center because in the Jackson, MS center only Black participants were recruited.

b Adjusted for age (continuous); race/center; female/not-current hormone replacement therapy user, female/current hormone replacement therapy user, male (ref); current smoker, former smoker, never smoker (ref); packyears (continuous plus a missing indicator); diagnosed diabetes, undiagnosed diabetes, at-risk for diabetes, normoglycemic (ref); BMI (continuous); at least some college, completed high school/equivalent, less than completed high school (ref) (Visit 1); current drinker, former drinker, never drinker (ref); hypertension status (yes/no); HDL cholesterol (continuous).

c Visit 2 (baseline) quartiles defined in the cohort used in the cancer analyses.

d From a Wald test of an interaction term between continuous IGFBP7 and age (continuous), race, or sex. Model 2 with the race or sex interaction terms with IGFBP7 does not include center or hormone replacement therapy, respectively.

**Table 3.** Association between plasma IGFBP7 RFU and risk of total cancer mortality, ARIC, Visit 2 to 2015.

	Cancer deaths	Person-years	Model 1^a^ HR and 95% CI	Model 2^b^ HR and 95% CI
Quartile^c^				
1	268	59177	reference	reference
2	362	56850	**1.24 (1.06 to 1.46)**	1.17 (0.99 to 1.37)
3	362	55203	**1.22 (1.03 to 1.43)**	1.08 (0.91 to 1.27)
4	428	51096	**1.59 (1.35 to 1.86)**	**1.27 (1.08 to 1.50)**
*P*-trend			**<.0001**	**.02**
Per doubling	1,420	222326	**1.87 (1.51 to 2.33)**	**1.37 (1.08 to 1.75)**
Stratified by age				
< 52.5y	228	60891	**2.72 (1.56 to 4.72)**	**1.97 (1.05 to 3.68)**
52.5 to ≤ 57.1y	308	58606	**2.01 (1.29 to 3.13)**	1.24 (0.75 to 2.07)
> 57.1 to 62.3y	415	54455	**1.88 (1.30 to 2.73)**	**1.60 (1.05 to 2.44)**
> 62.3y	469	48374	1.44 (0.95 to 2.20)	1.07 (0.69 to 1.66)
*P*-interaction^d^			.18	.17
Stratified by race				
White	1055	171602	**1.92 (1.50 to 2.46)**	**1.37 (1.03 to 1.81)**
Black	365	50724	**1.80 (1.15 to 2.81)**	1.40 (0.88 to 2.25)
*P*-interaction^d^			.98	.94
Stratified by sex				
Male	771	96073	**1.81 (1.33 to 2.45)**	**1.48 (1.06 to 2.06)**
Female	649	126253	**2.04 (1.49 to 2.79)**	1.32 (0.93 to 1.87)
*P*-interaction^d^			.80	.80

Bold denotes statistically significant.

a Adjusted for age (continuous); race/center (Black/Jackson, MS, Black/Other County, White/Forsyth County, NC, White/Washington County, MD, White/Minneapolis, MN [ref]); and sex (female/male).

b Adjusted for age (continuous); race/center; female/not-current hormone replacement therapy user, female/current hormone replacement therapy user, male (ref); current smoker, former smoker, never smoker (ref); packyears (continuous plus a missing indicator); diagnosed diabetes, undiagnosed diabetes, at-risk for diabetes, normoglycemic (ref); BMI (continuous); at least some college, completed high school/equivalent, less than completed high school (ref) (Visit 1); current drinker, former drinker, never drinker (ref); hypertension status (yes/no); HDL cholesterol (continuous).

c Visit 2 (baseline) quartiles defined in the cohort used in the cancer analyses.

d From a Wald test of an interaction term between continuous IGFBP7 and age (continuous), race, or sex. Model 2 with the race or sex interaction terms with IGFBP7 does not include center or hormone replacement therapy, respectively.

**Table 4.** The association between plasma IGFBP7 RFU and the risk of all-cause mortality, ARIC, Visit 2 to 2015.

	Deaths	Person-years	Model 1^a^ HR and 95% CI	Model 2^b^ HR and 95% CI
Quartile^c^				
1	1406	70915	reference	reference
2	1689	67395	**1.15 (1.07 to 1.24)**	**1.08 (1.00 to 1.16)**
3	1825	65074	**1.27 (1.18 to 1.37)**	**1.13 (1.05 to 1.22)**
4	2061	59344	**1.64 (1.52 to 1.76)**	**1.33 (1.23 to 1.43)**
*P*-trend			**<.0001**	**<.0001**
Per doubling	6981	262729	**2.22 (2.02 to 2.44)**	**1.67 (1.50 to 1.86)**
Stratified by age				
< 52.5y	981	74618	**3.54 (2.74 to 4.58)**	**2.52 (1.88 to 3.38)**
52.5 to ≤ 57.1y	1433	70164	**2.23 (1.82 to 2.72)**	**1.47 (1.16 to 1.88)**
> 57.1 to 62.3y	2014	63381	**2.07 (1.74 to 2.45)**	**1.71 (1.41 to 2.08)**
> 62.3y	2553	54567	**2.05 (1.73 to 2.45)**	**1.54 (1.28 to 1.86)**
*P*-interaction^d^			**.002**	**.0007**
Stratified by race				
White	5160	205021	**2.31 (2.08 to 2.58)**	**1.71 (1.51 to 1.94)**
Black	1821	57708	**2.04 (1.68 to 2.49)**	**1.71 (1.38 to 2.12)**
*P*-interaction^d^			.42	.61
Stratified by sex				
Male	3460	110779	**1.99 (1.73 to 2.29)**	**1.58 (1.35 to 1.85)**
Female	3521	151950	**2.52 (2.21 to 2.90)**	**1.83 (1.57 to 2.13)**
*P*-interaction^d^			.05	**.04**

Bold denotes statistically significant.

a Adjusted for age (continuous); race/center (Black/Jackson, MS, Black/Other County, White/Forsyth County, NC, White/Washington County, MD, White/Minneapolis, MN [ref]); and sex (female/male).

b Adjusted for age (continuous); race/center; female/not-current hormone replacement therapy user, female/current hormone replacement therapy user, male (ref); current smoker, former smoker, never smoker (ref); packyears (continuous plus a missing indicator); diagnosed diabetes, undiagnosed diabetes, at-risk for diabetes, normoglycemic (ref); BMI (continuous); at least some college, completed high school/equivalent, less than completed high school (ref) (Visit 1); current drinker, former drinker, never drinker (ref); hypertension status (yes/no); HDL cholesterol (continuous).

c Visit 2 (baseline) quartiles defined in the cohort used in the all-cause mortality analyses.

d From a Wald test of an interaction term between continuous IGFBP7 and age (continuous), race, or sex. Model 2 with the race or sex interaction terms with IGFBP7 does not include center or hormone replacement therapy, respectively.

## Data Availability

ARIC data, including the data used in this analysis, can be accessed by the following policy https://aric.cscc.unc.edu/aric9/researchers/Obtain_Submit_Data. ARIC data also are available via BioLINCC (controlled access database). Further information is available from the corresponding author upon request.

## References

[1] Orenduff MC, Pieper CF, Allott EH, Plasma insulin-like growth factor-binding protein-7 is positively associated with age, obesity, mortality, and cancer in postmenopausal women. Cancer Epidemiol Biomarkers Prev. 2025;34:922–932. 10.1158/1055-9965.EPI-24-1644 PMC12724625 40152978

[2] Meessen JMTA, Cesaroni G, Mureddu GF, IGFBP7 and GDF-15, but not P1NP, are associated with cardiac alterations and 10-year outcome in an elderly community-based study. BMC Cardiovasc Disord. 2021;21:328. 10.1186/s12872-021-02138-8 PMC8254994 34217226

[3] Gadd DA, Hillary RF, Kuncheva Z, Blood protein assessment of leading incident diseases and mortality in the UK Biobank. Nat Aging. 2024;4:939–948. 10.1038/s43587-024-00655-7 PMC11257969 38987645

[4] Papier K, Atkins JR, Tong TYN, Identifying proteomic risk factors for cancer using prospective and exome analyses of 1463 circulating proteins and risk of 19 cancers in the UK Biobank. Nat Commun. 2024;15:4010. 10.1038/s41467-024-48017-6 PMC11096312 38750076

[5] Knuppel A, Fensom GK, Watts EL, Circulating insulin-like growth factor-I concentrations and risk of 30 cancers: prospective analyses in UK biobank. Cancer Res. 2020;80:4014–4021. 10.1158/0008-5472.CAN-20-1281 32709735

[6] Murphy N, Knuppel A, Papadimitriou N, Insulin-like growth factor-1, insulin-like growth factor-binding protein-3, and breast cancer risk: observational and Mendelian randomization analyses with approximately 430 000 women. Ann Oncol. 2020;31:641–649. 10.1016/j.annonc.2020.01.066 PMC7221341 32169310

[7] Watts EL, Perez-Cornago A, Fensom GK, Circulating insulin-like growth factors and risks of overall, aggressive and early-onset prostate cancer: a collaborative analysis of 20 prospective studies and Mendelian randomization analysis. Int J Epidemiol. 2023;52:71–86. 10.1093/ije/dyac124 PMC9908067 35726641

[8] Akaogi K, Okabe Y, Sato J, Specific accumulation of tumor-derived adhesion factor in tumor blood vessels and in capillary tube-like structures of cultured vascular endothelial cells. Proc Natl Acad Sci USA. 1996;93:8384–8389. 10.1073/pnas.93.16.8384 PMC38680 8710880

[9] Evdokimova V, Tognon CE, Benatar T, IGFBP7 binds to the IGF-1 receptor and blocks its activation by insulin-like growth factors. Sci Signal. 2012;5:ra92. 10.1126/scisignal.2003184 23250396

[10] Xu Y, Sun Y, Zhu Y, Song G. Structural insight into CD93 recognition by IGFBP7. Structure. 2024;32:282–291.e4. 10.1016/j.str.2023.12.011 PMC12364601 38218180

[11] Godina C, Rosendahl AH, Goncalves de Oliveira K, Genetic determinants and clinical significance of circulating and tumor-specific levels of insulin-like growth factor binding protein 7 (IGFBP7) in a Swedish breast cancer cohort. Carcinogenesis. 2025;46:bgaf020. 10.1093/carcin/bgaf020 PMC12066007 40230015

[12] Wright JD, Folsom AR, Coresh J, The ARIC (Atherosclerosis Risk In Communities) study: JACC focus seminar 3/8. J Am Coll Cardiol. 2021;77:2939–2959. 10.1016/j.jacc.2021.04.035 PMC8667593 34112321

[13] Walker KA, Chen J, Zhang J, Large-scale plasma proteomic analysis identifies proteins and pathways associated with dementia risk. Nat Aging. 2021;1:473–489. 10.1038/s43587-021-00064-0 PMC10154040 37118015

[14] Rooney MR, Chen J, Ballantyne CM, Correlations within and between highly multiplexed proteomic assays of human plasma. Clin Chem. 2025;71:677–687. 10.1093/clinchem/hvaf030 PMC12129588 40172053

[15] Joshu CE, Barber JR, Coresh J, Enhancing the infrastructure of the Atherosclerosis Risk in Communities (ARIC) Study for Cancer Epidemiology Research: ARIC cancer. Cancer Epidemiol Biomarkers Prev. 2018;27:295–305. 10.1158/1055-9965.EPI-17-0696 PMC5835193 29263187

[16] Ru M, Douville C, Guenoun A, A smoking-related plasma protein score and smoking-related cancer risk and mortality in the Atherosclerosis Risk in Communities study. J Natl Cancer Inst. 2026;118:917–925. 10.1093/jnci/djag004 PMC12818548 41499436

[17] Sathyan S, Ayers E, Gao T, Plasma proteomic profile of age, health span, and all-cause mortality in older adults. Aging Cell. 2020;19:e13250. 10.1111/acel.13250 PMC7681045 33089916

[18] Shah AM, Myhre PL, Arthur V, Large scale plasma proteomics identifies novel proteins and protein networks associated with heart failure development. Nat Commun. 2024;15:528. 10.1038/s41467-023-44680-3 PMC10789789 38225249

[19] Guvatova ZG, Kobelyatskaya AA, Kudasheva ER, Matrisome transcriptome dynamics during tissue aging. Life (Basel). 2024;14:593. 10.3390/life14050593 PMC11121957 38792614

[20] Ding Y, Zuo Y, Zhang B, Comprehensive human proteome profiles across a 50-year lifespan reveal aging trajectories and signatures. Cell 2025;188:5763–5784.e26. 10.1016/j.cell.2025.06.047 40713952

[21] Severino V, Alessio N, Farina A, Insulin-like growth factor binding proteins 4 and 7 released by senescent cells promote premature senescence in mesenchymal stem cells. Cell Death Dis. 2013;4:e911. 10.1038/cddis.2013.445 PMC3847322 24201810

[22] Meyre PB, Aeschbacher S, Blum S, Biomarker panels for improved risk prediction and enhanced biological insights in patients with atrial fibrillation. Nat Commun. 2025;16:7042. 10.1038/s41467-025-62218-7 PMC12313968 40744929

[23] Aboumsallem JP, de Boer RA. IGFBP7: from senescence biomarker to a vaccine for heart failure. Circulation. 2024;150:390–392. 10.1161/CIRCULATIONAHA.124.067059 39074184

[24] Matarneh A, Akkari A, Sardar S, Beyond creatinine: diagnostic accuracy of emerging biomarkers for AKI in the ICU - a systematic review. Ren Fail. 2025;47:2556295. 10.1080/0886022X.2025.2556295 PMC12456049 40983596

[25] Wang Z, Burk VA, Huang Z, Identifying plasma proteins associated with risk of solid cancers: a 25-year prospective analysis of 4,712 circulating proteins in the ARIC study. medRxiv. 2026. 10.64898/2026.03.16.26348527

[26] Pitteri SJ, Hanash SM, Aragaki A, Postmenopausal estrogen and progestin effects on the serum proteome. Genome Med. 2009;1:121. 10.1186/gm121 PMC2808737 20034393

[27] Lit KK, Zhirenova Z, Blocki A. Insulin-like growth factor-binding protein 7 (IGFBP7): a microenvironment-dependent regulator of angiogenesis and vascular remodeling. Front Cell Dev Biol. 2024;12:1421438. 10.3389/fcell.2024.1421438 PMC11263173 39045455

[28] Xu Q, Wang J. IGFBP7 aggravates sepsis-induced acute lung injury by activating the ERK1/2 pathway. Folia Histochem Cytobiol. 2020;58:247–254. 10.5603/FHC.a2020.0028 33326113

[29] Zhao N, Ruan M, Koestler DC, Epigenome-wide scan identifies differentially methylated regions for lung cancer using pre-diagnostic peripheral blood. Epigenetics. 2022;17:460–472. 10.1080/15592294.2021.1923615 PMC8993104 34008478

[30] Arbiser JL. Diablo: a double-edged sword in cancer? Mol Ther. 2018;26:678–679. 10.1016/j.ymthe.2018.02.004 PMC5911152 29472100

