# Supplementary material for: Plasma insulin-like growth factor-binding protein 7 predicts 20-year cancer-specific and overall survival in the Atherosclerosis Risk in Communities Study
Source: J Natl Cancer Inst. Author manuscript; Available in PMC 2026 Sep 21. (PMC13591646; doi:10.1093/jnci/djag238)
Supplement: Supplementary file 1 [file NIHMS2208413-supplement-supplementary_material.docx]

| **Table S1.** Baseline Age-Adjusted Characteristics by Plasma IGFBP7 RFU Top and Bottom 5% Percentiles ^1^, ARIC, Visit 2 (N=11,761) | | | |
| --- | --- | --- | --- |
| Characteristics | Plasma IGFBP7 | | |
|  | Bottom 5%  Mean RFU (Range): 14.41 (13.48-14.57) | Middle 90%  Mean RFU (Range): 14.96 (14.57-15.31) | Top 5%  Mean RFU (Range): 15.50 (15.31-17.42) |
| Participants, N | 589 | 10,583 | 589 |
| Age, mean (SE), y | 56.2 (0.2) | 57.1 (0.1) | 58.2 (0.2) |
| Female, (%) | 93.7 | 54.2 | 42.4 |
| Black, (%) | 14.4 | 23.9 | 25.2 |
| Education level, (%) |  |  |  |
| Less than completed high school | 13.8 | 21.2 | 26.0 |
| Completed high school/equivalent | 46.3 | 42.5 | 41.0 |
| At least some college | 39.9 | 36.3 | 33.0 |
| Cigarette smoking status, (%) |  |  |  |
| Current | 12.9 | 22.3 | 32.0 |
| Former | 36.8 | 39.0 | 40.6 |
| Never | 50.3 | 38.7 | 27.4 |
| Packyears (ever smokers), mean (SE), y ^2^ | 21.2 (1.4) | 28.1 (0.3) | 36.3 (1.1) |
| BMI, mean (SE), kg/m^2^ | 26.6 (0.2) | 27.9 (0.1) | 28.9 (0.2) |
| Hypertension, (%) | 27.0 | 29.6 | 36.4 |
| Diabetes status, (%) |  |  |  |
| Diagnosed | 4.5 | 8.9 | 20.9 |
| Undiagnosed | 2.6 | 7.2 | 13.5 |
| At-risk | 39.5 | 49.6 | 41.3 |
| Normoglycemic | 53.4 | 34.3 | 24.3 |
| Alcohol drinking status, (%) |  |  |  |
| Current | 56.8 | 56.9 | 53.2 |
| Former | 15.8 | 20.8 | 29.8 |
| Never | 27.4 | 22.3 | 17.0 |
| Cholesterol, mean (SE), mg/dL |  |  |  |
| Total | 213.4 (1.6) | 210.6 (0.4) | 196.1 (1.6) |
| High density lipoprotein (HDL) | 62.4 (0.7) | 49.3 (0.2) | 45.6 (0.7) |
| Low density lipoprotein (LDL) | 119.4 (1.5) | 134.8 (0.4) | 122.2 (1.5) |
| Aspirin use (%) | 60.4 | 50.5 | 52.3 |
| HRT use (females), (%) |  |  |  |
| Current user | 75.2 | 11.6 | 3.8 |
| ^1^ Visit 2 (baseline) percentiles defined in the cohort used in the all-cause mortality analyses  ^2^ Of 7,222 ever smokers, packyears could not be determined for 635 (8.8%). They are excluded from Supplement Table 1 packyears estimates. | | | |

| **Table S2**. Association Between Plasma IGFBP7 RFU and Risk of Incident Site-Specific Cancers, ARIC, Visit 2 to 2015 | | | | |
| --- | --- | --- | --- | --- |
|  | Cancer Cases | Person-Years | Model 1 **^1^** | Model 2 **^2^** |
|  |  |  | HR and 95% CI | HR and 95% CI |
| **Breast Cancer Incidence** |  |  |  |  |
| Quartile ^3^ |  |  |  |  |
| 1 | 117 | 30,223 | reference | reference |
| 2 | 120 | 30,025 | 1.04 (0.81, 1.35) | 1.09 (0.84, 1.43) |
| 3 | 117 | 28,840 | 1.06 (0.82, 1.38) | 1.11 (0.84, 1.46) |
| 4 | 117 | 26,641 | 1.16 (0.89, 1.50) | 1.22 (0.92, 1.62) |
| p-trend |  |  | 0.7 | 0.6 |
| Per doubling | 471 | 115,729 | 1.13 (0.78, 1.65) | 1.21 (0.81, 1.82) |
|  |  |  |  |  |
| **Prostate Cancer Incidence** |  |  |  |  |
| Quartile ^3^ |  |  |  |  |
| 1 | 179 | 21,495 | reference | reference |
| 2 | 175 | 21,133 | 0.97 (0.79, 1.20) | 0.97 (0.79, 1.20) |
| 3 | 163 | 21,101 | 0.92 (0.75, 1.14) | 0.93 (0.75, 1.15) |
| 4 | 154 | 20,204 | 0.91 (0.74, 1.14) | 0.93 (0.75, 1.16) |
| p-trend |  |  | 0.8 | 0.9 |
| Per doubling | 671 | 83,933 | 0.86 (0.59, 1.27) | 0.89 (0.61, 1.31) |
|  |  |  |  |  |
|  | Cancer Cases | Person-Years | Model 1 **^1^** | Model 2 **^4^** |
|  |  |  | HR and 95% CI | HR and 95% CI |
| **Lung Cancer Incidence** |  |  |  |  |
| Quartile ^3^ |  |  |  |  |
| 1 | 76 | 53,428 | reference | reference |
| 2 | 114 | 50,982 | **1.39 (1.03, 1.86)** | 1.20 (0.89, 1.62) |
| 3 | 138 | 49,450 | **1.62 (1.21, 2.16)** | 1.25 (0.93, 1.69) |
| 4 | 151 | 45,964 | **1.90 (1.42, 2.53)** | 1.20 (0.89, 1.62) |
| p-trend |  |  | **0.0001** | 0.3 |
| Per doubling | 479 | 199,821 | **2.39 (1.71, 3.35)** | 1.42 (0.93, 2.15) |
|  |  |  |  |  |
|  | Cancer Cases | Person-Years | Model 1 **^1^** | Model 2 **^5^** |
|  |  |  | HR and 95% CI | HR and 95% CI |
| **Colorectal Cancer Incidence** |  |  |  |  |
| Quartile ^3^ |  |  |  |  |
| 1 | 77 | 53,428 | reference | reference |
| 2 | 78 | 50,982 | 0.92 (0.67, 1.27) | 0.95 (0.69, 1.33) |
| 3 | 77 | 49,449 | 0.88 (0.64, 1.23) | 0.90 (0.64, 1.26) |
| 4 | 80 | 45,962 | 1.00 (0.72, 1.38) | 0.97 (0.69, 1.37) |
| p-trend |  |  | 0.8 | 0.9 |
| Per doubling | 312 | 199,821 | 0.83 (0.50, 1.38) | 0.80 (0.47, 1.36) |
| Bold denotes statistically significant.  ^1^ Adjusted for Age (continuous); Race/Center (Black/Jackson, MS, Black/Other County, White/Forsyth County, NC, White/Washington County, MD, White/Minneapolis, MN (ref)); and Sex (female/male; Lung and Colorectal)  ^2^ Adjusted for Age (continuous); Race/Center; Current Hormone Replacement Therapy User, Not-Current Hormone Replacement Therapy User (ref) (Breast Cancer Analysis only); Current Smoker, Former Smoker, Never Smoker (ref); Packyears (continuous plus a missing indicator); Diagnosed Diabetes, Undiagnosed Diabetes, At-Risk for Diabetes, Normoglycemic (ref); BMI (continuous); At Least Some College, Completed High School/Equivalent, Less than Completed High School (ref) (Visit 1); Current Drinker, Former Drinker, Never Drinker (ref); Hypertension Status (yes/no); HDL Cholesterol (continuous)  ^3^ Visit 2 (baseline) quartiles defined in the cohort used in the cancer analyses  ^4^ Adjusted for Age (continuous); Race/Center; Female/Not-Current Hormone Replacement Therapy User, Female/Current Hormone Replacement Therapy User, Male (ref); Current Smoker, Former Smoker, Never Smoker (ref); Packyears (continuous plus a missing indicator); Diagnosed Diabetes, Undiagnosed Diabetes, At-Risk for Diabetes, Normoglycemic (ref); BMI (continuous); At Least Some College, Completed High School/Equivalent, Less than Completed High School (ref) (Visit 1); Current Drinker, Former Drinker, Never Drinker (ref); Hypertension Status (yes/no); HDL Cholesterol (continuous); In the fully-adjusted model, after adjusting for the smoking score, the Q4 vs. Q1 HR was 1.15 (95% CI 0.85-1.56). Spearman correlation between IGFBP7 and score was r=0.19.  ^5^ Adjusted for Age (continuous); Race/Center; Female/Not-Current Hormone Replacement Therapy User, Female/Current Hormone Replacement Therapy User, Male (ref); Current Smoker, Former Smoker, Never Smoker (ref); Packyears (continuous plus a missing indicator); Diagnosed Diabetes, Undiagnosed Diabetes, At-Risk for Diabetes, Normoglycemic (ref); BMI (continuous); At Least Some College, Completed High School/Equivalent, Less than Completed High School (ref) (Visit 1); Current Drinker, Former Drinker, Never Drinker (ref); Hypertension Status (yes/no); HDL Cholesterol (continuous) | | | | |

| **Table S3**. Association Between Plasma IGFBP7 RFU and Risk of Site-Specific Cancer Mortality, ARIC, Visit 2 to 2015 | | | | |
| --- | --- | --- | --- | --- |
|  | Cancer Deaths | Person-Years | Model 1 **^1^** | Model 2 **^2^** |
|  |  |  | HR and 95% CI | HR and 95% CI |
| **Breast Cancer Mortality** |  |  |  |  |
| Quartile ^3^ |  |  |  |  |
| 1 | 14 | 33,172 | reference | reference |
| 2 | 24 | 32,588 | 1.65 (0.85, 3.19) | 1.44 (0.73, 2.84) |
| 3 | 25 | 31,304 | 1.68 (0.87, 3.25) | 1.33 (0.66, 2.64) |
| 4 | 28 | 29,033 | **2.08 (1.09, 3.98)** | 1.61 (0.81, 3.21) |
| p-trend |  |  | 0.2 | 0.6 |
| Per doubling | 91 | 126,097 | **2.43 (1.07, 5.52)** | 1.69 (0.66, 4.35) |
|  |  |  |  |  |
| **Prostate Cancer Mortality** |  |  |  |  |
| Quartile ^3^ |  |  |  |  |
| 1 | 23 | 24,931 | reference | reference |
| 2 | 19 | 24,201 | 0.82 (0.45, 1.51) | 0.82 (0.44, 1.50) |
| 3 | 15 | 24,146 | 0.69 (0.36, 1.31) | 0.70 (0.36, 1.34) |
| 4 | 21 | 22,796 | 1.06 (0.58, 1.93) | 1.01 (0.55, 1.85) |
| p-trend |  |  | 0.6 | 0.6 |
| Per doubling | 78 | 96,074 | 1.44 (0.51, 4.07) | 1.42 (0.50, 4.07) |
|  |  |  |  |  |
|  |  |  | Model 1 **^1^** | Model 2 **^4^** |
|  |  |  | HR and 95% CI | HR and 95% CI |
| **Lung Cancer Mortality** |  |  |  |  |
| Quartile ^3^ |  |  |  |  |
| 1 | 58 | 59,178 | reference | reference |
| 2 | 100 | 56,851 | **1.52 (1.10, 2.11)** | 1.34 (0.96, 1.87) |
| 3 | 114 | 55,203 | **1.66 (1.20, 2.30)** | 1.29 (0.92, 1.80) |
| 4 | 145 | 51,096 | **2.29 (1.67, 3.14)** | **1.48 (1.07, 2.05)** |
| p-trend |  |  | **<0.0001** | **0.04** |
| Per doubling | 417 | 222,329 | **2.74 (1.94, 3.89)** | **1.66 (1.07, 2.57)** |
|  |  |  |  |  |
|  |  |  | Model 1 **^1^** | Model 2 **^5^** |
|  |  |  | HR and 95% CI | HR and 95% CI |
| **Colorectal Cancer Mortality** |  |  |  |  |
| Quartile ^3^ |  |  |  |  |
| 1 | 28 | 59,178 | reference | reference |
| 2 | 32 | 56,851 | 1.00 (0.60, 1.68) | 1.05 (0.62, 1.79) |
| 3 | 27 | 55,203 | 0.82 (0.47, 1.41) | 0.84 (0.48, 1.47) |
| 4 | 29 | 51,096 | 0.96 (0.56, 1.65) | 0.89 (0.50, 1.57) |
| p-trend |  |  | 0.9 | 0.8 |
| Per doubling | 116 | 222,329 | 0.71 (0.31, 1.66) | 0.64 (0.26, 1.54) |
| ^1^ Adjusted for Age (continuous); Race/Center (Black/Jackson, MS, Black/Other County, White/Forsyth County, NC, White/Washington County, MD, White/Minneapolis, MN (ref)); and Sex (female/male; Lung and Colorectal)  ^2^ Adjusted for Age (continuous); Race/Center; Current Hormone Replacement Therapy User, Not-Current Hormone Replacement Therapy User (ref) (Breast Cancer Analysis only); Current Smoker, Former Smoker, Never Smoker (ref); Packyears (continuous plus a missing indicator); Diagnosed Diabetes, Undiagnosed Diabetes, At-Risk for Diabetes, Normoglycemic (ref); BMI (continuous); At Least Some College, Completed High School/Equivalent, Less than Completed High School (ref) (Visit 1); Current Drinker, Former Drinker, Never Drinker (ref); Hypertension Status (yes/no); HDL Cholesterol (continuous)  ^3^ Visit 2 (baseline) quartiles defined in the cohort used in the cancer analyses  ^4^ Adjusted for Age (continuous); Race/Center; Female/Not-Current Hormone Replacement Therapy User, Female/Current Hormone Replacement Therapy User, Male (ref); Current Smoker, Former Smoker, Never Smoker (ref); Packyears (continuous plus a missing indicator); Diagnosed Diabetes, Undiagnosed Diabetes, At-Risk for Diabetes, Normoglycemic (ref); BMI (continuous); At Least Some College, Completed High School/Equivalent, Less than Completed High School (ref) (Visit 1); Current Drinker, Former Drinker, Never Drinker (ref); Hypertension Status (yes/no); HDL Cholesterol (continuous). In the fully-adjusted model, after adjusting for the smoking score, the Q4 vs. Q1 HR was 1.42 (95% CI 1.02-1.97). Spearman correlation between IGFBP7 and score was r=0.19.  ^5^ Adjusted for Age (continuous); Race/Center; Female/Not-Current Hormone Replacement Therapy User, Female/Current Hormone Replacement Therapy User, Male (ref); Current Smoker, Former Smoker, Never Smoker (ref); Packyears (continuous plus a missing indicator); Diagnosed Diabetes, Undiagnosed Diabetes, At-Risk for Diabetes, Normoglycemic (ref); BMI (continuous); At Least Some College, Completed High School/Equivalent, Less than Completed High School (ref) (Visit 1); Current Drinker, Former Drinker, Never Drinker (ref); Hypertension Status (yes/no); HDL Cholesterol (continuous) | | | | |

| **Table S4**. Association Between Plasma IGFBP7 RFU and Risk of Total Cancer Mortality, ARIC, Analyses Truncated at 18-20, 13-15, 8-10, and 3-5 years of follow-up time, Visit 2 to 2010, 2005, 2000, and 1995 | | | | |
| --- | --- | --- | --- | --- |
|  | Cancer Deaths | Person-Years | Model 1 **^1^** | Model 2 **^2^** |
|  |  |  | HR and 95% CI | HR and 95% CI |
| **18-20 years of follow-up (Visit 2 to 12/31/2010)** | | | | |
| Quartile ^3^ |  |  |  |  |
| 1 | 193 | 48,963 | reference | reference |
| 2 | 282 | 47,747 | **1.30 (1.08, 1.57)** | **1.21 (1.00, 1.47)** |
| 3 | 247 | 46,710 | 1.10 (0.91, 1.34) | 0.97 (0.79, 1.18) |
| 4 | 332 | 43,857 | **1.59 (1.32, 1.92)** | **1.25 (1.04, 1.52)** |
| p-trend |  |  | **<0.0001** | **0.004** |
| Per doubling | 1,054 | 187,278 | **1.80 (1.40, 2.31)** | 1.28 (0.97, 1.69) |
|  |  |  |  |  |
| **13-15 years of follow-up (Visit 2 to 12/31/2005)** | | | | |
| Quartile ^3^ |  |  |  |  |
| 1 | 123 | 37,465 | reference | reference |
| 2 | 171 | 36,951 | 1.22 (0.96, 1.54) | 1.12 (0.88, 1.42) |
| 3 | 153 | 36,322 | 1.05 (0.82, 1.33) | 0.90 (0.70, 1.16) |
| 4 | 219 | 34,752 | **1.57 (1.25, 1.97)** | 1.20 (0.94, 1.52) |
| p-trend |  |  | **<0.0001** | 0.05 |
| Per doubling | 666 | 145,490 | **1.69 (1.23, 2.33)** | 1.14 (0.80, 1.62) |
|  |  |  |  |  |
| **8-10 years of follow-up (Visit 2 to 12/31/2000)** | | | | |
| Quartile ^3^ |  |  |  |  |
| 1 | 60 | 25,035 | reference | reference |
| 2 | 79 | 24,852 | 1.13 (0.81, 1.59) | 0.98 (0.70, 1.38) |
| 3 | 85 | 24,557 | 1.15 (0.82, 1.61) | 0.93 (0.66, 1.31) |
| 4 | 129 | 23,974 | **1.78 (1.30, 2.45)** | 1.28 (0.93, 1.77) |
| p-trend |  |  | **0.0003** | 0.09 |
| Per doubling | 353 | 98,417 | **1.94 (1.27, 2.95)** | 1.24 (0.77, 2.00) |
|  |  |  |  |  |
| **3-5 years of follow-up (Visit 2 to 12/31/1995)** | | | | |
| Quartile ^3^ |  |  |  |  |
| 1 | 26 | 11,988 | reference | reference |
| 2 | 28 | 11,919 | 0.96 (0.56, 1.65) | 0.80 (0.47, 1.39) |
| 3 | 33 | 11,787 | 1.08 (0.64, 1.84) | 0.83 (0.48, 1.41) |
| 4 | 54 | 11,761 | **1.74 (1.07, 2.84)** | 1.17 (0.71, 1.93) |
| p-trend |  |  | **0.02** | 0.3 |
| Per doubling | 141 | 47,455 | **2.00 (1.04, 3.84)** | 1.21 (0.57, 2.58) |
| Bold denotes statistically significant.  ^1^ Adjusted for Age (continuous); Race/Center (Black/Jackson, MS, Black/Other County, White/Forsyth County, NC, White/Washington County, MD, White/Minneapolis, MN (ref)); and Sex (female/male)  ^2^ Adjusted for Age (continuous); Race/Center; Female/Not-Current Hormone Replacement Therapy User, Female/Current Hormone Replacement Therapy User, Male (ref); Current Smoker, Former Smoker, Never Smoker (ref); Packyears (continuous plus a missing indicator); Diagnosed Diabetes, Undiagnosed Diabetes, At-Risk for Diabetes, Normoglycemic (ref); BMI (continuous); At Least Some College, Completed High School/Equivalent, Less than Completed High School (ref) (Visit 1); Current Drinker, Former Drinker, Never Drinker (ref); Hypertension Status (yes/no); HDL Cholesterol (continuous)  ^3^ Visit 2 (baseline) quartiles defined in the cohort used in the cancer analyses | | | | |

| **Table S5**. Association Between Plasma IGFBP7 RFU and Risk of Total Cancer Mortality, ARIC, Analyses Lagged by 5 and 10 years, (1995-1997), (2000-2002) to 2015 | | | | |
| --- | --- | --- | --- | --- |
|  | Cancer  Deaths | Person-Years | Model 1 **^1^** | Model 2 **^2^** |
|  |  |  | HR and 95% CI | HR and 95% CI |
| **Lagged by 5 years** | | | | |
| Quartile ^3^ |  |  |  |  |
| 1 | 214 | 43,681 | reference | reference |
| 2 | 298 | 41,687 | **1.29 (1.08, 1.54)** | **1.22 (1.01, 1.46)** |
| 3 | 294 | 40,726 | **1.23 (1.03, 1.48)** | 1.10 (0.91, 1.32) |
| 4 | 339 | 38,163 | **1.53 (1.28, 1.83)** | **1.25 (1.03, 1.50)** |
| p-trend |  |  | **<0.0001** | 0.07 |
| Per doubling | 1,145 | 164,257 | **1.83 (1.44, 2.33)** | **1.37 (1.04, 1.79)** |
|  |  |  |  |  |
| **Lagged by 10 years** | | | | |
| Quartile ^3^ |  |  |  |  |
| 1 | 147 | 29,005 | reference | reference |
| 2 | 199 | 27,874 | 1.24 (1.00, 1.53) | 1.14 (0.91, 1.42) |
| 3 | 207 | 27,025 | **1.26 (1.02, 1.57)** | 1.11 (0.89, 1.39) |
| 4 | 238 | 25,947 | **1.51 (1.22, 1.87)** | 1.21 (0.97, 1.52) |
| p-trend |  |  | **0.003** | 0.4 |
| Per doubling | 791 | 109,850 | **1.88 (1.41, 2.51)** | **1.41 (1.02, 1.94)** |
| Bold denotes statistically significant.  ^1^ Adjusted for Age (continuous); Race/Center (Black/Jackson, MS, Black/Other County, White/Forsyth County, NC, White/Washington County, MD, White/Minneapolis, MN (ref)); and Sex (female/male)  ^2^ Adjusted for Age (continuous); Race/Center; Female/Not-Current Hormone Replacement Therapy User, Female/Current Hormone Replacement Therapy User, Male (ref); Current Smoker, Former Smoker, Never Smoker (ref); Packyears (continuous plus a missing indicator); Diagnosed Diabetes, Undiagnosed Diabetes, At-Risk for Diabetes, Normoglycemic (ref); BMI (continuous); At Least Some College, Completed High School/Equivalent, Less than Completed High School (ref) (Visit 1); Current Drinker, Former Drinker, Never Drinker (ref); Hypertension Status (yes/no); HDL Cholesterol (continuous)  ^3^ Visit 2 (baseline) quartiles defined in the cohort used in the cancer analyses | | | | |

| **Table S6**. Association Between Plasma IGFBP7 RFU and Risk of All-Cause Mortality, ARIC, Analyses Truncated at 18-20, 13-15, 8-10, and 3-5 years of follow-up, Visit 2 through 2015, 2010, 2005, 2000, and 1995 | | | | |
| --- | --- | --- | --- | --- |
|  | Deaths | Person-  years | Model 1 **^1^** | Model 2 **^2^** |
|  |  |  | HR and 95% CI | HR and 95% CI |
| **23-25 years of follow-up (Visit 2 to 12/31/2015)** | | | | |
| Quartile ^3^ |  |  |  |  |
| 1 | 927 | 62,832 | reference | reference |
| 2 | 1,171 | 60,468 | **1.17 (1.07, 1.27)** | 1.09 (0.99, 1.19) |
| 3 | 1,338 | 58,842 | **1.32 (1.21, 1.44)** | **1.15 (1.05, 1.26)** |
| 4 | 1,603 | 54,242 | **1.76 (1.62, 1.91)** | **1.38 (1.27, 1.51)** |
| p-trend |  |  | **<0.0001** | **<0.0001** |
| Per doubling | 5,039 | 236,384 | **2.40 (2.16, 2.68)** | **1.77 (1.57, 2.00)** |
|  |  |  |  |  |
| **18-20 years of follow-up (Visit 2 to 12/31/2010)** | | | | |
| Quartile ^3^ |  |  |  |  |
| 1 | 593 | 52,326 | reference | reference |
| 2 | 808 | 51,074 | **1.22 (1.09, 1.35)** | **1.13 (1.01, 1.26)** |
| 3 | 887 | 50,035 | **1.29 (1.16, 1.44)** | **1.13 (1.01, 1.26)** |
| 4 | 1,203 | 46,786 | **1.90 (1.72, 2.11)** | **1.47 (1.32, 1.63)** |
| p-trend |  |  | **<0.0001** | **<0.0001** |
| Per doubling | 3,491 | 200,222 | **2.60 (2.30, 2.94)** | **1.91 (1.66, 2.20)** |
|  |  |  |  |  |
| **13-15 years of follow-up (Visit 2 to 12/31/2005)** | | | | |
| Quartile ^3^ |  |  |  |  |
| 1 | 348 | 40,176 | reference | reference |
| 2 | 466 | 39,705 | **1.16 (1.01, 1.33)** | 1.04 (0.90, 1.20) |
| 3 | 539 | 39,034 | **1.28 (1.12, 1.47)** | 1.07 (0.93, 1.24) |
| 4 | 807 | 37,212 | **2.01 (1.76, 2.28)** | **1.47 (1.28, 1.68)** |
| p-trend |  |  | **<0.0001** | **<0.0001** |
| Per doubling | 2,160 | 156,127 | **2.92 (2.51, 3.40)** | **2.06 (1.73, 2.47)** |
|  |  |  |  |  |
| **8-10 years of follow-up (Visit 2 to 12/31/2000)** | | | | |
| Quartile ^3^ |  |  |  |  |
| 1 | 184 | 26,937 | reference | reference |
| 2 | 221 | 26,819 | 1.03 (0.85, 1.26) | 0.89 (0.73, 1.09) |
| 3 | 287 | 26,474 | **1.27 (1.05, 1.54)** | 1.03 (0.85, 1.24) |
| 4 | 463 | 25,777 | **2.09 (1.76, 2.50)** | **1.47 (1.23, 1.76)** |
| p-trend |  |  | **<0.0001** | **<0.0001** |
| Per doubling | 1,155 | 106,008 | **3.37 (2.78, 4.09)** | **2.43 (1.93, 3.07)** |
|  |  |  |  |  |
| **3-5 years of follow-up (Visit 2 to 12/31/1995)** | | | | |
| Quartile ^3^ |  |  |  |  |
| 1 | 74 | 12,956 | reference | reference |
| 2 | 79 | 12,897 | 0.94 (0.68, 1.30) | 0.79 (0.57, 1.09) |
| 3 | 114 | 12,759 | 1.30 (0.97, 1.76) | 1.02 (0.76, 1.38) |
| 4 | 181 | 12,676 | **2.05 (1.55, 2.71)** | **1.40 (1.06, 1.86)** |
| p-trend |  |  | **<0.0001** | **0.0002** |
| Per doubling | 448 | 51,289 | **3.40 (2.51, 4.60)** | **2.44 (1.69, 3.51)** |
| Bold denotes statistically significant.  ^1^ Adjusted for Age (continuous); Race/Center (Black/Jackson, MS, Black/Other County, White/Forsyth County, NC, White/Washington County, MD, White/Minneapolis, MN (ref)); and Sex (female/male)  ^2^ Adjusted for Age (continuous); Race/Center; Female/Not-Current Hormone Replacement Therapy User, Female/Current Hormone Replacement Therapy User, Male (ref); Current Smoker, Former Smoker, Never Smoker (ref); Packyears (continuous plus a missing indicator); Diagnosed Diabetes, Undiagnosed Diabetes, At-Risk for Diabetes, Normoglycemic (ref); BMI (continuous); At Least Some College, Completed High School/Equivalent, Less than Completed High School (ref) (Visit 1); Current Drinker, Former Drinker, Never Drinker (ref); Hypertension Status (yes/no); HDL Cholesterol (continuous)  ^3^ Visit 2 (baseline) quartiles defined in the cohort used in the all-cause mortality analyses | | | | |

| **Table S7**. Association Between Plasma IGFBP7 RFU and Risk of All-Cause Mortality, ARIC, Analyses Lagged by 5 and 10 years, (1995-1997), (2000-2002) to 2020 | | | | |
| --- | --- | --- | --- | --- |
|  | Deaths | Person-years | Model 1 **^1^** | Model 2 **^2^** |
|  |  |  | HR and 95% CI | HR and 95% CI |
| Lagged by 5 years |  |  |  |  |
| Quartile ^3^ |  |  |  |  |
| 1 | 1,304 | 55,672 | reference | reference |
| 2 | 1,572 | 52,218 | **1.17 (1.08, 1.26)** | **1.09 (1.01, 1.18)** |
| 3 | 1,696 | 50,659 | **1.27 (1.18, 1.37)** | **1.14 (1.05, 1.23)** |
| 4 | 1,898 | 46,579 | **1.59 (1.48, 1.71)** | **1.30 (1.21, 1.40)** |
| p-trend |  |  | **<0.0001** | **<0.0001** |
| Per doubling | 6,470 | 205,128 | **2.12 (1.91, 2.34)** | **1.60 (1.43, 1.80)** |
|  |  |  |  |  |
| Lagged by 10 years |  |  |  |  |
| Quartile ^3^ |  |  |  |  |
| 1 | 1,159 | 40,756 | reference | reference |
| 2 | 1,390 | 38,270 | **1.15 (1.06, 1.24)** | 1.08 (0.99, 1.17) |
| 3 | 1,505 | 36,997 | **1.27 (1.17, 1.37)** | **1.15 (1.06, 1.25)** |
| 4 | 1,677 | 34,859 | **1.52 (1.41, 1.65)** | **1.27 (1.17, 1.37)** |
| p-trend |  |  | **<0.0001** | **<0.0001** |
| Per doubling | 5,731 | 150,882 | **1.97 (1.77, 2.20)** | **1.52 (1.34, 1.71)** |
| Bold denotes statistically significant.  ^1^ Adjusted for Age (continuous); Race/Center (Black/Jackson, MS, Black/Other County, White/Forsyth County, NC, White/Washington County, MD, White/Minneapolis, MN (ref)); and Sex (female/male)  ^2^ Adjusted for Age (continuous); Race/Center; Female/Not-Current Hormone Replacement Therapy User, Female/Current Hormone Replacement Therapy User, Male (ref); Current Smoker, Former Smoker, Never Smoker (ref); Packyears (continuous plus a missing indicator); Diagnosed Diabetes, Undiagnosed Diabetes, At-Risk for Diabetes, Normoglycemic (ref); BMI (continuous); At Least Some College, Completed High School/Equivalent, Less than Completed High School (ref) (Visit 1); Current Drinker, Former Drinker, Never Drinker (ref); Hypertension Status (yes/no); HDL Cholesterol (continuous)  ^3^ Visit 2 (baseline) quartiles defined in the cohort used in the all-cause mortality analyses | | | | |

| **Table S8**. Association Between Plasma IGFBP7 RFU and Risk of All-Cause Mortality After Excluding Prevalent Heart Failure and Censoring for Incident Heart Failure, ARIC, Visit 2 to 2020 ^1^ | | | | |
| --- | --- | --- | --- | --- |
|  | Deaths | Person-  years | Model 1 **^2^** | Model 2 **^3^** |
|  |  |  | HR and 95% CI | HR and 95% CI |
| Quartile ^4^ |  |  |  |  |
| 1 | 874 | 63,974 | reference | reference |
| 2 | 1,032 | 59,908 | **1.17 (1.07, 1.28)** | **1.10 (1.00, 1.21)** |
| 3 | 1,072 | 57,036 | **1.25 (1.14, 1.37)** | **1.14 (1.04, 1.25)** |
| 4 | 1,147 | 50,779 | **1.57 (1.43, 1.72)** | **1.31 (1.20, 1.45)** |
| p-trend |  |  | **<0.0001** | **<0.0001** |
| Per doubling | 4,125 | 231,698 | **1.96 (1.72, 2.23)** | **1.53 (1.32, 1.76)** |
| Bold denotes statistically significant.  ^1^ 813 participants were excluded due to prevalent heart failure before Visit 2 or missing data related to heart failures  ^2^ Adjusted for Age (continuous); Race/Center (Black/Jackson, MS, Black/Other County, White/Forsyth County, NC, White/Washington County, MD, White/Minneapolis, MN (ref)); and Sex (female/male)  ^3^ Adjusted for Age (continuous); Race/Center; Female/Not-Current Hormone Replacement Therapy User, Female/Current Hormone Replacement Therapy User, Male (ref); Current Smoker, Former Smoker, Never Smoker (ref); Packyears (continuous plus a missing indicator); Diagnosed Diabetes, Undiagnosed Diabetes, At-Risk for Diabetes, Normoglycemic (ref); BMI (continuous); At Least Some College, Completed High School/Equivalent, Less than Completed High School (ref) (Visit 1); Current Drinker, Former Drinker, Never Drinker (ref); Hypertension Status (yes/no); HDL Cholesterol (continuous)  ^4^ Visit 2 (baseline) quartiles defined in the cohort used in the all-cause mortality analyses | | | | |

| **Table S9**. Association Between Plasma IGFBP7 RFU and Risk of All-Cause Mortality, ARIC, 5 years of follow-up starting at Visit 5 time | | | | |
| --- | --- | --- | --- | --- |
|  | Deaths | Person-years | Model 1 **^1^** | Model 2 **^2^** |
|  |  |  | HR and 95% CI | HR and 95% CI |
| **IGFBP7 measured at start of follow-up (Visit 5)** | | | | |
| Quartile ^3^ |  |  |  |  |
| 1 | 110 | 5,035 | reference | reference |
| 2 | 120 | 5,024 | 1.08 (0.83, 1.40) | 1.07 (0.82, 1.38) |
| 3 | 132 | 5,000 | 1.18 (0.91, 1.52) | 1.11 (0.86, 1.43) |
| 4 | 219 | 4,805 | **1.99 (1.58, 2.51)** | **1.90 (1.50, 2.39)** |
| p-trend |  |  | **<0.0001** | **<0.0001** |
| Per doubling | 581 | 19,864 | **2.71 (2.09, 3.51)** | **2.57 (1.96, 3.38)** |
|  |  |  |  |  |
|  | Deaths | Person-years | Model 1 **^4^** | Model 2 **^5^** |
|  |  |  | HR and 95% CI | HR and 95% CI |
| **IGFBP7 measured 18 years before start of follow-up (Visit 3)** | | | | |
| Quartile ^3^ |  |  |  |  |
| 1 | 107 | 5,033 | reference | reference |
| 2 | 146 | 4,966 | 1.28 (1.00, 1.65) | 1.27 (0.98, 1.63) |
| 3 | 147 | 4,984 | 1.18 (0.92, 1.53) | 1.11 (0.86, 1.43) |
| 4 | 181 | 4,881 | **1.49 (1.17, 1.91)** | 1.28 (1.00, 1.65) |
| p-trend |  |  | **0.01** | 0.2 |
| Per doubling | 581 | 19,864 | **2.02 (1.44, 2.84)** | **1.60 (1.12, 2.29)** |
|  |  |  |  |  |
|  | Deaths | Person-years | Model 1 **^6^** | Model 2 **^7^** |
|  |  |  | HR and 95% CI | HR and 95% CI |
| **IGFBP7 measured 21 years before the start of follow-up (Visit 2)** | | | | |
| Quartile ^3^ |  |  |  |  |
| 1 | 111 | 5,021 | reference | reference |
| 2 | 146 | 5,010 | 1.17 (0.91, 1.50) | 1.10 (0.86, 1.42) |
| 3 | 142 | 4,946 | 1.09 (0.84, 1.41) | 0.99 (0.77, 1.28) |
| 4 | 182 | 4,888 | **1.40 (1.09, 1.79)** | 1.14 (0.89, 1.47) |
| p-trend |  |  | **0.03** | 0.5 |
| Per doubling | 581 | 19,864 | **1.81 (1.28, 2.57)** | 1.32 (0.90, 1.93) |
| Bold denotes statistically significant.  ^1^ Adjusted for Age (continuous) (Visit 5); Race/Center (Black/Jackson, MS, Black/Other County, White/Forsyth County, NC, White/Washington County, MD, White/Minneapolis, MN (ref)); and Sex (female/male)  ^2^ Adjusted for Age (continuous) (Visit 5); Race/Center; Female/Not-Current Hormone Replacement Therapy User, Female/Current Hormone Replacement Therapy User, Male (ref) (Visit 5); Current Smoker, Former Smoker, Never Smoker (ref) (Visit 5); Packyears (continuous plus a missing indicator) (Visit 5); Diagnosed Diabetes, Undiagnosed Diabetes, At-Risk for Diabetes, Normoglycemic (ref) (Visit 5); BMI (continuous) (Visit 5); At Least Some College, Completed High School/Equivalent, Less than Completed High School (ref) (Visit 1); Current Drinker, Former Drinker, Never Drinker (ref) (Visit 5); Hypertension Status (yes/no) (Visit 5); HDL Cholesterol (continuous) (Visit 5)  ^3^ Visit 2 (baseline) quartiles defined in the cohort used in the all-cause mortality analyses  ^4^ Adjusted for Age (continuous) (Visit 3); Race/Center (Black/Jackson, MS, Black/Other County, White/Forsyth County, NC, White/Washington County, MD, White/Minneapolis, MN (ref)); and Sex (female/male)  ^5^ Adjusted for Age (continuous) (Visit 3); Race/Center; Female/Not-Current Hormone Replacement Therapy User, Female/Current Hormone Replacement Therapy User, Male (ref) (Visit 3); Current Smoker, Former Smoker, Never Smoker (ref) (Visit 3); Packyears (continuous plus a missing indicator) (Visit 3); Diagnosed Diabetes, Undiagnosed Diabetes, At-Risk for Diabetes, Normoglycemic (ref) (Visit 3); BMI (continuous) (Visit 3); At Least Some College, Completed High School/Equivalent, Less than Completed High School (ref) (Visit 1); Current Drinker, Former Drinker, Never Drinker (ref) (Visit 3); Hypertension Status (yes/no) (Visit 3); HDL Cholesterol (continuous) (Visit 3)  ^6^ Adjusted for Age (continuous); Race/Center (Black/Jackson, MS, Black/Other County, White/Forsyth County, NC, White/Washington County, MD, White/Minneapolis, MN (ref)); and Sex (female/male)  ^7^ Adjusted for Age (continuous); Race/Center; Female/Not-Current Hormone Replacement Therapy User, Female/Current Hormone Replacement Therapy User, Male (ref); Current Smoker, Former Smoker, Never Smoker (ref); Packyears (continuous plus a missing indicator); Diagnosed Diabetes, Undiagnosed Diabetes, At-Risk for Diabetes, Normoglycemic (ref); BMI (continuous); At Least Some College, Completed High School/Equivalent, Less than Completed High School (ref) (Visit 1); Current Drinker, Former Drinker, Never Drinker (ref); Hypertension Status (yes/no); HDL Cholesterol (continuous) | | | | |

| **Table S10.** Top Spearman correlation coefficients between IGFBP7 and aptamers in the SomaScan panel, ARIC, Visit 2^1^ | |
| --- | --- |
| **SomaScan Protein** | **r** |
| Diablo homolog, mitochondrial | 0.56 |
| Calsyntenin-2 | 0.45 |
| Olfactomedin-like protein 3 | 0.41 |
| Netrin-4 | 0.41 |
| Polypeptide N-acetylgalactosaminyltransferase 16 | 0.37 |
| GDNF family receptor alpha-1 | 0.36 |
| Testican-2 | 0.35 |
| Pregnancy zone protein | -0.35 |
| Receptor-type tyrosine-protein phosphatase U | 0.35 |
| Angiotensinogen | -0.34 |
| ^1^ 10 proteins with the strongest correlations with Plasma IGFBP7 at Visit 2 among 4,955 aptamers in the SomaScan panel; all p<0.0001 | |

| **Table S11.** The Association Between Plasma IGFBP7 RFU and the Risk of All-Cause Mortality, Adjusted for Top Correlated Proteins^1^, ARIC, Visit 2 - 2020 | | |
| --- | --- | --- |
|  | Model 1 ^2, 4^ | Model 2 ^3, 4^ |
|  | HR^4^ and 95% CI | HR^4^ and 95% CI |
| Without Adjustment for Correlated Proteins (Table) | 2.22 (2.02, 2.44) | 1.67 (1.50, 1.86) |
| *Adjusted for:* | | |
| Diablo homolog, mitochondrial | 2.01 (1.82, 2.23) | 1.54 (1.38, 1.73) |
| Calsyntenin-2 | 2.07 (1.86, 2.29) | 1.58 (1.40, 1.77) |
| Olfactomedin-like protein 3 | 2.21 (2.00, 2.44) | 1.69 (1.51, 1.89) |
| Netrin-4 | 1.91 (1.72, 2.12) | 1.53 (1.37, 1.72) |
| Polypeptide N-acetylgalactosaminyltransferase 16 | 2.21 (2.00, 2.45) | 1.62 (1.45, 1.82) |
| GDNF family receptor alpha-1 | 2.13 (1.92, 2.35) | 1.64 (1.47, 1.84) |
| Testican-2 | 2.27 (2.06, 2.50) | 1.72 (1.53, 1.92) |
| Pregnancy zone protein | 2.29 (2.08, 2.52) | 1.69 (1.52, 1.89) |
| Receptor-type tyrosine-protein phosphatase U | 1.99 (1.80, 2.21) | 1.65 (1.47, 1.84) |
| Angiotensinogen | 2.16 (1.95, 2.39) | 1.69 (1.51, 1.88) |
| ^1^ Selected the top 10 proteins with the strongest correlations with Plasma IGFBP7 at Visit 2 (See Supplement Table 10)  ^2^ Adjusted for Age (continuous); Race/Center (Black/Jackson, MS, Black/Other County, White/Forsyth County, NC, White/Washington County, MD, White/Minneapolis, MN (ref)); and Sex (female/male)  ^3^ Adjusted for Age (continuous); Race/Center; Female/Not-Current Hormone Replacement Therapy User, Female/Current Hormone Replacement Therapy User, Male (ref); Current Smoker, Former Smoker, Never Smoker (ref); Packyears (continuous plus a missing indicator); Diagnosed Diabetes, Undiagnosed Diabetes, At-Risk for Diabetes, Normoglycemic (ref); BMI (continuous); At Least Some College, Completed High School/Equivalent, Less than Completed High School (ref) (Visit 1); Current Drinker, Former Drinker, Never Drinker (ref); Hypertension Status (yes/no); HDL Cholesterol (continuous) | | |
